# Severe *Plasmodium vivax* Malaria in Pakistan

**DOI:** 10.3201/eid1911.130495

**Published:** 2013-11

**Authors:** Ali Bin Sarwar Zubairi, Sobia Nizami, Afsheen Raza, Vikram Mehraj, Anita Fazal Rasheed, Najia Karim Ghanchi, Zahra Nur Khaled, M. Asim Beg

**Affiliations:** Aga Khan University, Karachi, Pakistan (A.B.S. Zubairi, S. Nizami, A. Raza, A.F. Rasheed, N.K. Ghanchi, Z.N. Khaled, M.A. Beg);; Aix Marseille Université, Marseille, France (V. Mehraj)

**Keywords:** malaria, severe malaria, complications, Plasmodium vivax, Pakistan, parasites

## Abstract

To compare the severity of *Plasmodium vivax* malaria with that of *P. falciparum* malaria, we conducted a retrospective cross-sectional study of 356 adults hospitalized with malaria (2009–2011) in Pakistan. *P. vivax* and *P. falciparum* accounted for 83% and 13% of cases, respectively; 79.9% of patients with severe malaria were infected with *P. vivax.*

Malaria is endemic to Pakistan and 64% and 36% of malaria cases are attributed to *Plasmodium vivax* and *P. falciparum*, respectively (*1*). The purpose of this study was to identify the complications of *P. vivax* among hospitalized malaria patients and compare the prevalence of these complications with those of *P. falciparum* malaria.

## The Study

We conducted a retrospective cross-sectional study using convenience sampling at the Aga Khan University Hospital in Karachi, Pakistan. Participants were all adult patients (>16 years of age) who were hospitalized with malaria during January 2009–December 2011. Reasons for hospitalization included intravenous antimalarial therapy, management of associated diagnoses, and complications. The following data on patients were retrieved through the hospital’s electronic and file records: age, sex, infecting *Plasmodium* species, malaria diagnosis methods, co-existing conditions, results of biochemical and microbiological investigations, radiographic findings, complications, hospital course, and outcome.

Records showed that Giemsa-stained peripheral blood smears, the malaria rapid diagnostic test (RDT), or both, were used for malaria diagnosis. The RDT used antibodies against *P. falciparum* histidine-rich protein 2 and *P. vivax* lactate dehydrogenase. For 45 case-patients for which results from peripheral blood smears and RDTs were discordant or unreliable, surface protein-specific PCR was performed by using stored patient blood samples to identify the *Plasmodium* species (*2*,*3*). Clinical syndromes were classified as severe on the basis of the World Health Organization’s 2010 severe *falciparum* malaria criteria (*4*).

Statistical analysis was performed by using SPSS version 20 (http://www-01.ibm.com/software/analytics/spss/). Averages, χ^2^ test of independence, odds ratios with 95% CIs, and analysis of variance were computed when applicable.

Case-patients with prior co-morbid conditions were excluded from relevant subanalyses, for example, diabetes mellitus patients were excluded from hypoglycemia analysis. All analysis was also repeated after excluding all case-patients with associated infections and comorbid illnesses. The classification “comorbidity” included all conditions in the Charlson comorbidity index for mortality (*5*). The study was approved by the Aga Khan University’s Ethics Review Committee.

**Figure 1:**
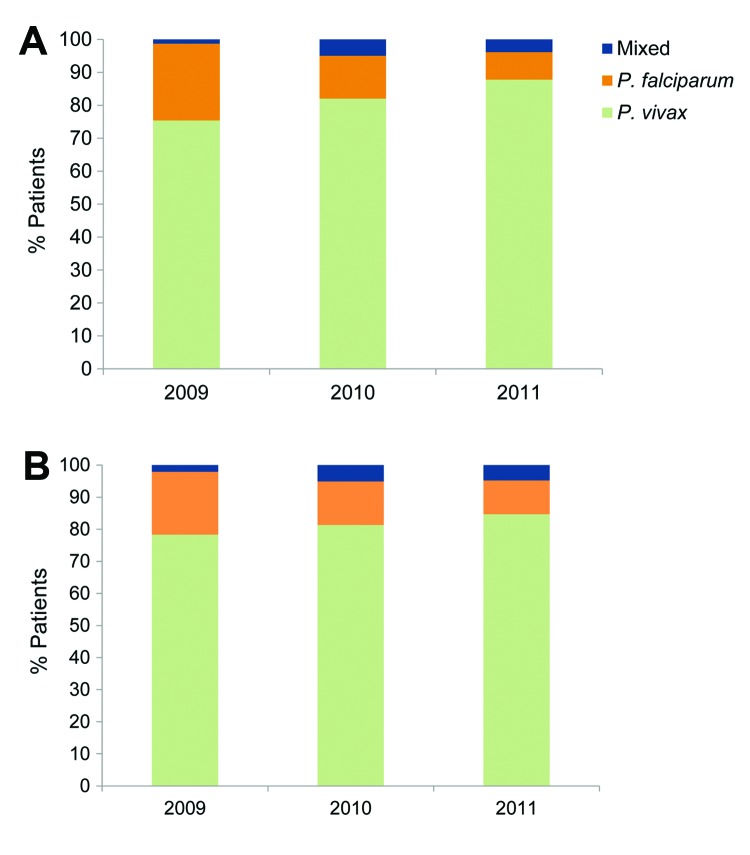
A) Proportion of hospitalized cases of *Plasmodium vivax* (n = 296), *P. falciparum* (n = 47), and mixed (n = 13) infections, Karachi, Pakistan, 2009–2011. B) Number of hospitalized cases of *P. vivax* (n = 189), *P. falciparum* (n = 30), and mixed (n = 10) infections, after excluding patients with concurrent illnesses, 2009–2011.

A total of 356 patients with malaria (mean ± SD age 42 ± 18 years) were hospitalized in the Aga Khan Hospital during 2009–2011. Among these, 296 (83.1%), 47 (13.2%), and 13 (3.7%) were found to have *P. vivax* infection, *P. falciparum* infection*,* and mixed infections ( *P. vivax* and *P. falciparum),* respectively. Baseline patient demographics are given in Table 1. The proportion of *P. vivax* infection among hospitalized malaria patients increased from 75.0% in 2009 to 87.7% in 2011 (p<0.02) (Figure 1, panel A).

**Table 1:** Demographic profile of study participants with *Plasmodium vivax* and *P. falciparum* malaria, Karachi, Pakistan, 2009–2011*

Characteristic	Frequency (%)		
	*P. vivax*	*P. falciparum*	Mixed
Sex			
F	98 (33)	12 (25)	6 (46)
M	198 (67)	35 (75)	7 (54)
Previously healthy adults	189 (64)	30 (64)	10 (77)
Concurrent illness			
Diabetes	49 (17)	4 (9)	0
Ischemic heart disease	37 (12)	2 (4)	3 (23)
Chronic kidney disease	10 (3)	3 (6)	0
Co-existing infection†	34 (12)	5 (11)	0
Others‡	10 (3)	5 (11)	0
Total§	107 (36)	17 (36)	3 (23)

*n = 356.  †Co-existing infections included dengue fever, urinary tract infection, enteric fever, and hepatitis C, diagnosed by appropriate serologic testing/culture. ‡Other conditions included chronic obstructive pulmonary disease, chronic liver disease, malignancy, and other conditions from the Charlson Comorbidity Index (*5*). §Many patients had multiple comorbidities; therefore, the total does not sum the above.

**Figure 2:**
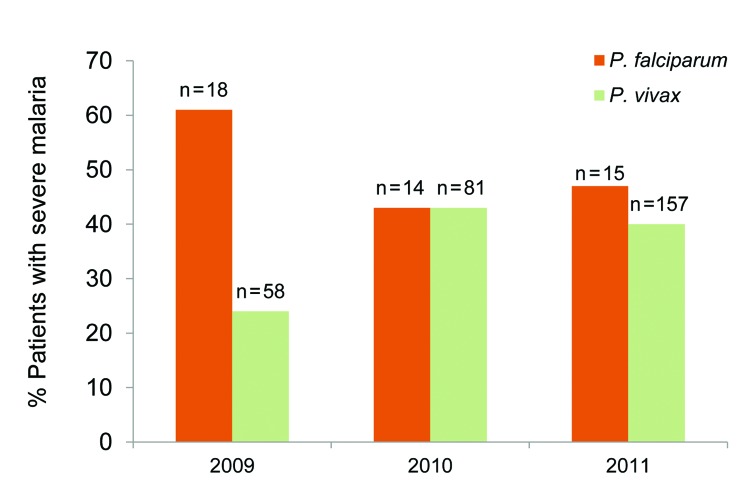
Percentage of *Plasmodium falciparum* and *P. vivax* patients with severe malaria, Karachi, Pakistan, 2009–2011. The number of mixed infections (n = 13) over 3 years was too small for comparison.

One hundred thirty-nine (39.0%) patients had at least 1 complication by World Health Organization criteria (*4*), among which 111 (79.9%) patients had *P. vivax* infection. In 24 (51.0%) cases of *P. falciparum* infections and in 111 cases (37.5%) of *P. vivax* infections, respectively, severe malaria developed (p = 0.077). As shown in Figure 2, the proportion of severe malaria among *P. vivax* patients increased from 24.1% in 2009 to 43.2% in 2010 and 39.5% in 2011 (p = 0.02).

The most common complications in the patients are shown in Table 2. *P. vivax* and *P. falciparum* were responsible for comparable rates of pulmonary edema, the need for mechanical ventilation, coagulopathy, hypoglycemia, hemoglobinuria, metabolic acidosis, renal impairment, liver dysfunction, bleeding, and multi-organ dysfunction. Altered consciousness, anemia, and jaundice were associated with *P. falciparum* malaria. The mean platelet count for *P. vivax* patients was 55, significantly lower than that of *P. falciparum* patients (67.5; p = 0.001) and those with mixed infections (61; p = 0.024).

**Table 2:** Comparison of complication rates in *P. falciparum* versus *P. vivax* infections, Karachi, Pakistan, 2009–20011*

Complications		Case definition	No. (%) *P. falciparum* cases; n = 47		No. (%) *P. vivax* cases; n = 296		Odds ratio (CI)			p value	
WHO criteria†										
Altered consciousness	Disorientation or confusion			5 (10.6)		6 (2.0)		5.7 (1.7–19.7)	0.002	
Metabolic acidosis	Plasma bicarbonate <15 mmol/L			5 (10.6)		17 (5.7)		1.9 (0.7–5.6)	0.203	
Pulmonary edema	Respiratory distress and bilateral diffuse infiltrates on chest radiograph			6 (12.8)		23 (7.8)		1.7 (0.7–4.5)	0.253	
Abnormal spontaneous bleeding	Bleeding from gastrointestinal, genitourinary or respiratory tracts			1 (2.1)		16 (5.4)		0.4 (0.049–2.9)	0.336	
Jaundice	Serum bilirubin >3.0 mg/dL			12 (25.5)		28(9.5)		3.3(1.5–7.0)	0.001	
Hemoglobinuria	Hemoglobin in urine			15 (31.9)		62 (20.9)		1.8 (0.9–3.4)	0.094	
Shock	Systolic blood pressure <80 mm Hg			4 (8.5)		5 (1.7)		5.4 (1.4–20.9)	0.007	
Hypoglycemia‡	Blood glucose <40 mg/dL			1 (2.1)		3 (1.0)		2.1 (0.2–20.9)	0.509	
Renal impairment§	Serum creatinine >3 mg/dL			2 (4.3)		10 (3.4)		1.3 (0.3–6.0)	0.761	
Other										
Hyperpyrexia	Core body temperature >40°C			4 (8.5)		32 (10.8)		0.8 (0.4–1.9)	0.416	
Thrombocytopenia	Platelets <150,000/mm^3^			39 (83.0)		272 (91.9)		0.4 (0.2–1.0)	0.051	
Profound	<20,000/mm^3^			5 (10.6)		58 (19.6)		0.5 (0.2–1.0)	0.141	
Anemia	Hemoglobin <7 mg/dL			10 (21.3)		15 (5.1)		5.0 (2.1–12.1)	0.000	
Multiorgan dysfunction	Biochemical and /or radiographic evidence of ≥2 organs involved			5 (10.6)		21 (7.1)		1.6 (0.6–4.4)	0.394	
Secondary infection	Radiographic/microbiological evidence of infection			9 (19.1)		2 (7.4)		2.9 (1.3–6.9)	0.009	
Coagulopathy	Deranged PT/APTT			5 (10.6)		17 (5.7)		2.0 (0.7–5.6)	0.203	
Liver dysfunction	ALT level >normal			16 (44.4)		97 (40.9)		1.1 (0.5–1.9)	0.690	

*WHO, World Health Organization; PT, prothrombin time; APTT, activated partial thromboplastin time. ALT, alanine aminotransferase. †Source: (*4*). ‡Patients with preexisting diabetes were excluded from this count; n = 303. §Patients with preexisting chronic kidney disease were excluded from this count; n = 343.

The mean hospital stay was 4.1 days for *P. falciparum* patients, 3.6 days for *P. vivax* patients, and 2.9 days for patients with mixed infections. Three *P. vivax* malaria patients experienced fatal acute myocardial infarctions. One patient, who had metastatic myeloma and *P. falciparum* malaria, died. The mortality rate was 2.1% for *P. falciparum* patients and 1.0% for *P. vivax* patients (p = 0.50).

Analysis was repeated after all patients with comorbid conditions were excluded (Table 1), which left 229 case-patients who had no illness other than malaria. Among these, 30 (13%) patients had *P. falciparum* infection, 189 (83%) had *P. vivax* infection, and 10 (4%) had mixed infection (Figure 1, panel B). In these patients, severe malaria appeared significantly more common in falciparum versus vivax malaria (53% and 33%, respectively, p = 0.029); however, 79.5% of the severe cases were caused by *P. vivax*. Hemoglobinuria and a higher mean creatinine level were more likely to occur with falciparum malaria than with vivax malaria (p<0.02). Shock and secondary bacterial infections were no longer associated with *P. falciparum* infection. All other statistical associations held, although the strength of association varied.

## Conclusions 

A study of hospitalized malaria patients at the Aga Khan University Hospital during 1997–2001 showed that 51.8% of cases were caused by *P. vivax* and 46.5% by *P. falciparum*, with mortality rates of 1.5% and 2.0%, respectively (*6*). Recent studies from elsewhere in Asia reported that 20%– 40% of patients hospitalized with malaria had *P. vivax* malaria (*7*), with mortality rates of 0.8%–1.6% (*7*). In our study, a much greater proportion of malaria cases were caused by *P. vivax* (83%), which was not unexpected because of the decreasing number of *P. falciparum* cases during the study period. Despite this high incidence of *P. vivax* malaria, the mortality rate found in our study is reassuring and stable at 1.0%.

The higher prevalence of jaundice, anemia, and hemoglobinuria seen with falciparum malaria in our study reflect the greater degree of hemolysis caused by *P. falciparum*. *P. vivax* has been reported elsewhere to cause a similar degree of anemia as *P. falciparum* (*8*). Differences in the level of endemic anemia between these study populations and may explain this discrepancy. Similar to our findings, another study reported the incidence of thrombocytopenia in hospitalized patients with vivax malaria as high as 96.3% (*9*). Pulmonary involvement has often been reported in complicated vivax malaria (*7*), as we found in our study. Hepatic dysfunction with jaundice has been reported in up to 57% of hospitalized *P. vivax* patients (*10*); our findings were similar.

To estimate the true effects of severe disease with vivax malaria, researchers have recommended excluding comorbid conditions (*7*) and other infections (*11*). In this study, excluding concurrent illness enabled a stronger association between *P. falciparum* and severe malaria to emerge. Thus, *P. falciparum* caused a higher likelihood of specific complications such as central nervous system disturbance and hemolysis than did *P. vivax*. Yet, ≈80% of severe malaria still occurred in patients with *P. vivax* malaria.

Limitations of the study include its retrospective design, low power, and lack of PCR diagnostics for all the samples. Furthermore, the study findings reflect the malaria situation at a single urban tertiary care hospital, which cannot be generalized without knowing the denominator of all hospitalized malaria cases in the study area.

*P. vivax* is a major contributor to the disease effects of malaria, including severe malaria, in a tertiary care setting in Karachi, Pakistan. Furthermore, *P. falciparum* and *P. vivax* have similar rates for several complications (pulmonary edema, metabolic acidosis, abnormal bleeding, renal impairment) and death.
